# Neuronal Mitochondrial Dysfunction Drives Astrocytic Mitochondrial Transfer after TBI: Reveals the Therapeutic Potential of astrocytic EV-Mito

**DOI:** 10.21203/rs.3.rs-10105855/v1

**Published:** 2026-07-06

**Authors:** Gopal V Velmurugan, Hemendra J Vekaria, Alexander G Rabchevsky, Kai Saito, Josh M Morganti, Samir Patel, Brad Hubbard, Patrick G Sullivan

**Affiliations:** University of Kentucky; University of Kentucky; University of Kentucky; University of Kentucky; University of Kentucky; University of Kentucky; University of Kentucky; University of Kentucky

**Keywords:** Intercellular mitochondrial transfer, cell-type specific mitochondria, TBI, excitotoxicity, extracellular vesicles containing mitochondria (EV-mito), exogenous mitochondrial transfer

## Abstract

Mitochondria are dynamic organelles essential for neuronal survival and synaptic function, and their dysfunction is a key consequence of excitotoxicity following traumatic brain injury (TBI). While intercellular mitochondrial transfer and exogenous mitochondrial transplantation have emerged as mechanisms to restore cellular bioenergetics, its *in vivo* relevance in the central nervous system remains incompletely understood. Here, we used astrocyte and neuron-specific mitochondrial reporters (GFP or Dendra2) in mice to assess cell-type-specific mitochondrial morphology, bioenergetics, and transfer 24hrs after TBI. Neurons exhibited marked mitochondrial dysfunction, including altered morphology and reduced bioenergetic capacity across somatic, synaptic, and non-neuronal fractions. In contrast, astrocytic mitochondria showed morphological changes but preserved bioenergetic function. Concomitantly, astrocyte-to-neuron mitochondrial transfer was significantly increased following injury, although transfer to synapses remained limited. Single-cell RNA sequencing of astrocytes revealed upregulation of genes involved in extracellular vesicle (EV) biogenesis and mitochondrial translation following injury compared to controls. *In vitro* co-culture studies confirmed that astrocytes transfer mitochondria to neurons via EVs containing mitochondria (EV-mito). Isolated EV-mito from astrocyte-conditioned media improves neuronal mitochondrial function under NMDA (N-methyl-D-aspartate) induced excitotoxic conditions. Together, these findings demonstrate that neuronal mitochondrial dysfunction drives astrocyte-mediated mitochondrial transfer as an adaptive neuroprotective response after TBI. This process preserves neuronal bioenergetics in the soma and neurites but not at synapses, highlighting both its therapeutic potential and spatial limitations.

## Introduction

Traumatic brain injury (TBI) is a leading cause of disability that affects millions of individuals each year in the United States [1]. People who sustain TBI often experience a wide range of persistent symptoms, including cognitive impairment, depression, and an elevated risk of developing dementia [2, 3]. Currently, there are no FDA-approved therapeutics that directly target the neurobiological consequences of TBI, and a substantial knowledge gap remains in our understanding of both the injury processes and their long-term sequelae [4].

Mitochondria are central players in neurobiological responses to brain trauma, including neuronal resilience. Mitochondrial dysfunction is a well-established hallmark of secondary injury after TBI [5–9]. One critical function of mitochondria is buffering excess calcium during glutamate-induced excitotoxicity, a process that particularly affects neurons [10]. Following trauma, elevations in cytosolic calcium can overload mitochondria and trigger the mitochondrial permeability transition (mPT) [11]. Once initiated, mPT causes mitochondria to release calcium back into the cytosol, setting off a cascade of permeability transition events that culminate in neuronal cell death [12]. One potential protective strategy is to increase the mitochondrial pool within neurons. A larger mitochondrial network increases the cell’s overall calcium buffering capacity, allowing the mitochondria to absorb and store more calcium before any single mitochondrion reaches the threshold for mPT pore opening. Intercellular horizontal mitochondrial transfer (IMT) is a process through which one cell donates mitochondria to another [13, 14], thereby representing a way to expand the neuronal mitochondrial pool in response to cellular insults such as TBI.

IMT has been documented in several organ systems to eliminate defective mitochondria [15, 16] and act as a signaling mechanism for cellular protection[17, 18]. IMT also occurs within the central nervous system (CNS) as a means of transmitophagy [19] and mitochondrial protection after injury [20]. Astrocytes are critical in the acute phase following TBI [21], and are thought to be the primary donor cell type of mitochondria in the CNS, supplying them to both neurons and endothelial cells [20, 22–24]. However, whether astrocytes donate mitochondria to neurons following TBI and how this process influences neuronal and astrocytic function remains unclear. Addressing this gap requires a better understanding of the cellular mechanisms that govern IMT in the injured brain. To date, IMT appears to occur through both contact-dependent routes, such as tunneling nanotubes (TNT), and contact-independent pathways, including extracellular vesicles (EV-mito) [25–27]. Astrocytes have been shown to release mitochondria packaged within EV (Ast-EV-mito) [22], raising the possibility that this process contributes to endogenous neuroprotection that could be harnessed therapeutically through exogenous mitochondrial transplantation (EMT).

Here, we tested the hypothesis that astrocytes transfer mitochondria to neurons following TBI to restore neuronal bioenergetics and function. Using astrocyte and neuron-specific mitochondrial reporter mice expressing mitochondrial-targeted GFP (mtGFP) or Dendra2 (mtD2), we characterized cell-specific mitochondrial morphology, bioenergetics, and intercellular mitochondrial transfer under physiological and injury conditions in vivo. We further investigated mitochondrial transfer from astrocytes and astrocytic derived EV-mito to neurons and evaluated the ability of EV-mito to rescue NMDA-induced excitotoxic mitochondrial dysfunction in primary cortical neurons. Collectively, these studies identify astrocytic mitochondrial transfer as an endogenous neuroprotective response to TBI and highlight the therapeutic potential of EV-mito mediated mitochondrial transplantation for CNS disorders associated with mitochondrial dysfunction.

## Materials and methods

### Experimental animals

All animal experiments were performed in accordance with the NIH Guide for the Care and Use of Laboratory Animals and were approved by the Institutional Animal Care and Use Committee at the University of Kentucky. All the breeder mice were purchased from Jackson Laboratories, bred and maintained at the Division of Laboratory Animal Resources (DLAR) at the University of Kentucky. Throughout the study, animals were socially housed in individually ventilated cages on a 12 hrs light cycle and had ad libitum access to food and water.

*Global mtD2 mice*: Mice (B6;129S-Gt (ROSA)26Sortm1.1 (CAG-COX8A/Dendra2) Dcc/J) expressing a mitochondrial-specific version of Dendra2 green (mtD2) was used to globally express mtD2 (Strain #018397). *Astrocytic mitochondrial reporter mice*: A tamoxifen inducible astrocyte-specific mitochondrial reporter mouse expressing mtD2 (AmtD2) targeted to the inner mitochondrial membrane (IMM) and GFP (AmtGFP) targeted to the outer mitochondrial membrane (OMM) were generated by crossing Mice B6;129S-Gt (ROSA)26Sortm1(CAG-COX8A/Dendra2) Dcc/J (Jax#018385) or B6N.Cg-Gt (ROSA)26Sortm1(CAG-EGFP/Synj2bp) Thm/J (Jax#032675) with B6N.FVB-Tg (Aldh1l1-cre/ERT2)1Khakh/J (Jax #031008), respectively. Cre-positive animals were injected with tamoxifen (TAM) at 75mg/kg body weight around 6-weeks of age for five consecutive days. One month after TAM injection, animals were used for experimental purposes. *Neuronal mitochondrial reporter mice*: Like astrocyte mitochondrial reporter mice, neuron-specific mitochondrial reporter mice were generated by crossing Jax#018385 (mtD2) and Jax#032675 (mtGFP) mice with B6.Cg-Tg(Camk2a-cre)T29–1Stl/J (Jax#005359). All animals were older than 3 months of age because CamK2a expression starts only around 2 months of age.

### Controlled cortical impact

Controlled cortical impact (CCI) was performed as previously described (1.0 mm depth of contusion at 3.5 m/s with a dwell time of 500 ms) [28–30]. In brief, animals were anaesthetized using isoflurane (2–5%), shaved, cleaned, and prepared for the surgical procedure. A Kopf stereotaxic frame was used to generate brain injury under a pneumatic impactor (Precision Science Instruments). A longitudinal skin incision was made down the middle of the head dorsum before craniotomy (4mm) was performed on the left side of the skull between lambda and bregma. Mice were injured by hitting the dura mater of the brain using a 3mm flat-tip impactor. The injury area was cleaned using cotton tips to mitigate any bleeding, the craniotomy was covered with surgiseal, and the wound was closed using staples.

#### Immunofluorescence:

Brain cryosection (all the mtGFP), paraffin sections (all the mtD2), and cultured primary cells were immunostained as described previously [30]. 10% Neutral buffered formalin (NBF)-fixed cultured cells and/or brain sections were permeabilized and blocked in phosphate-buffered saline-Tween 20 (PBST) + 0.2% Triton X-100 in + 1% BSA and 10% normal horse serum for one hour at RT. Then, the cells/sections were incubated overnight at 4°C with primary antibody (1:250 dilution; Table 1) in antibody dilution buffer (blocking buffer and PBST at a 1:1 ratio). This was followed by incubation with fluorophore-labeled secondary antibody (1:500 dilution; Table 1) at RT for 1 h. After washing, the cell cultures and/or sections were mounted on glass slides using Vectashield antifade mounting medium with or without DAPI (H-1500 and H-1400; Vector Laboratories, USA). For cells cultured on glass-bottom plates, cells were directly fixed, stained, and imaged without mounting.

### Confocal imaging and image processing

Fluorescence Z-stack images were acquired using confocal microscopes (Nikon A1R and AXR) equipped with 20× air and 100× oil immersion objectives. Image acquisition and processing were performed using NIS-Elements software (version 5.30.05). Z-stack images were denoised and, in some cases, deconvoluted to enhance resolution. As previously reported [30], 3D reconstructions were generated from Z-stack data using either NIS-Elements or Imaris software (version 10.2.0), to visualize mitochondrial transfer events between cells. For quantitative analysis, a custom pipeline was created using the General Analysis 3 (GA3) module in NIS-Elements (Fig. S3). This pipeline was employed to determine the total surface area of primary neurons and to quantify the number of astrocyte-derived mitochondria transferred into neurons. Neurons were defined as the region of interest (ROI) within 3D reconstructions for analyses.

### Primary cortical neuron culture

Primary cortical neurons were isolated from wild-type mouse embryos at embryonic days 13–15. Brain cells were isolated via enzymatic digestion as previously described, with slight modifications [22]. Cells were cultured on poly-D-lysine-coated glass-bottom 24-well plates (Fisher Scientific; A3890401; VWR; 82050898). Briefly, after removal of the meninges, cortical tissues were dissected, minced, and digested in 0.05% trypsin for 15–20 minutes at 37°C with gentle agitation every 5 minutes. Digestion was halted using DMEM supplemented with 10% fetal bovine serum (FBS), followed by gentle trituration (~ 15–20 passes) to dissociate the tissue. The suspension was allowed to settle for 1 minute to remove debris, and the supernatant was then passed through a 70 μm cell strainer. Cells were pelleted by centrifugation at 300 × g for 5 minutes at room temperature and resuspended in high-glucose DMEM supplemented with 10% FBS and antibiotics (ThermoFisher; 15-140-122). Cells were plated and maintained in the initial culture medium for 12 hours, after which the medium was replaced with neurobasal medium supplemented with B-27 and GlutaMAX. Cells were cultured either in 96-well Seahorse XF culture plates (~ 25–30,000) for mitochondrial stress test (MST) assays at DIV 8–13.

### Primary astrocyte culture and astrocytic EV-mito enrichment

Primary cortical astrocyte cultures were derived from 1- to 3-day-old postnatal global mtD2-expressing pups, following the same digestion protocol as described above, with the exception that only astrocyte culture medium with supplements was obtained from ScienceCell (MSPP-1801). Upon reaching confluency (8–10 days), primary astrocytes were isolated by first removing nonadherent glial cells through orbital shaking at 200 rpm for 6 hours. Then, the astrocytes were dissociated using trypsinization and sub-cultured for further experiments. After 4 days, the primary astrocyte-conditioned media were centrifuged at 1000 g for 10 minutes to remove cell debris, and the supernatant was centrifuged at 13,000 g for 30 minutes at 4 °C to enrich astrocytic EV-mito.

### Neuron-astrocyte and neuron-EV-mito co-culture

Human neuroblastoma cells (SH-SY5Y) were co-cultured with primary astrocytes expressing mtD2 (AmtD2 cells) in glass-bottom slides for 3–4 days or with enriched astrocyte-derived EV-mito for 24–48hrs. To detect mitochondrial transfer from astrocytes or EV-mito to neurons, cells were stained with Mitotracker Deep Red FM (500nM) (Thermo Fisher; M22426) for 20 minutes and Hoechst dye (1 μg/ml) for 5 minutes at 37°C. Cells were washed with warm media at least 3 times to remove excess dye, and live cells were imaged under a confocal microscope at 100 and 40X oil.

### Tetramethylrhodamine, ethyl ester (TMRE) staining

Isolated mitochondria were incubated with 500 nM TMRE in respiration buffer containing pyruvate, malate, and ADP for 10 minutes at room temperature. Excess dye was removed by washing prior to imaging.

#### Astrocytic EV-mito treatment and Mitochondrial stress test (MST):

Primary cortical neurons (~ 25–30,000 cells/well) were treated with NMDA (100 μM) or vehicle for 12 hours. Cells were then washed and subsequently incubated for 24hrs with astrocyte-derived EV-mito isolated from conditioned media collected from five confluent T-75 flasks (50 ml of media), as described above and resulting EV-mito pellet was resuspended and used to treat 18 wells of primary neurons seeded in a 96-well Seahorse XF cell culture microplate. MST was performed on a Seahorse XFe96 Flux Analyzer (Agilent Technologies, Palo Alto, CA, USA) according to the manufacturer’s instructions. Briefly, the cells were incubated in XF assay medium supplemented with substrates (1 mM pyruvate and 2 mM L-glutamine) for 1hr before the oxygen consumption rate (OCR) measurement. After three measurements of baseline OCR, respiratory chain inhibitors/uncouplers were added sequentially into each well as follows: 1 μM Oligomycin, 4 μM FCCP, and 0.5 μM Rotenone/Antimycin. After each injection, an additional three OCR readings were taken. Different OCR parameters were calculated by the Wave software version (Agilent Technologies). Final OCR measurements were normalized to the number of cells. The Experiment was repeated 2 times with 5–10 technical replicates for each treatment condition.

#### Western blot:

Protein lysate was prepared from isolated mitochondrial fractions using RIPA buffer (150 mM NaCl, 1% Triton X-100, 0.5% sodium deoxycholate, 0.1% SDS, 50 mM Tris, pH 8.0) and centrifuged at 16,100× g for 30 min. Total protein levels were estimated in the supernatant using a BCA kit (23225, Thermofisher). Western blot samples were obtained using XT sample buffer (1610791, Biorad, Hercules, CA, USA) with DTT and boiled at 95°C for 10 min. The samples (6 μg protein) were resolved in a 4–12% BIS-TRIS gel (3450125, Bio-Rad, Hercules, CA, USA) under reducing conditions and transferred to a nitrocellulose membrane. Probing was performed using CD63 (1:1000; NBP2-42225, Novus Biologicals) and beta-Actin (1:2000; 8H10D10; Cell Signaling). The signals were detected using IRDy 700RD goat anti-rabbit and 680RD goat anti-mouse (1: 10,000; 926-32211 and 926-68070, Li-Cor, Lincoln, NE, USA). The protein levels were quantified with densitometric analysis of the Western blot bands using ImageJ software.

### Cell-specific fractionated mitochondrial magnetic separation:

#### Neuronal mitochondrial fractions:

Purified neuronal mitochondrial fractions were obtained from NmtGFP mice using magnetic bead-based immunoisolation with modifications to previously described protocols [31]. Briefly, brain tissue (cortex, hippocampus, or pooled regions) was homogenized in mitochondrial isolation buffer (IB) using a Dounce homogenizer (8–10 strokes). Homogenates were centrifuged at 1,300 × g for 3 min at 4°C, and the supernatant was collected. At this stage, small aliquots were reserved as the total mitochondrial fraction for use as controls. The remaining supernatant was incubated with anti-GFP antibody (25 μL bead per 20 mg of brain tissue) in a total volume of 10 mL per LS MACS column (Miltenyi). Following 20 min of rotation at 4°C, the suspension was applied to the column. The flow-through (elute) contained synaptosomes, whereas the retained fraction represented the somatic mitochondrial pool that was recovered by flushing the column with 1.5 mL IB, followed by centrifugation (13,000 × g, 10 min, 4°C). Pellets were resuspended in IB, protein concentrations quantified, and aliquots prepared for downstream assays. The synaptosome-containing elute was subsequently centrifuged (13,000 × g, 10 min, 4°C) and pellets were resuspended in 500 μL IB. Synaptosomes were disrupted using a nitrogen bomb (1,200 psi, 10 min) to release synaptic mitochondria. The lysate was then incubated with anti-GFP antibody–conjugated beads (20 min, rotation), passed through a fresh LS column, and separated into two fractions: (i) non-neuronal (NN) mitochondria, recovered from the column flow-through and (ii) synaptic mitochondria, eluted by flushing the column with 1.5 mL IB. Both synaptic and NN fractions were centrifuged at 13,000 × g for 10 min at 4°C, pellets were resuspended in IB, protein quantified and used for downstream applications.

### Astrocytic mitochondrial fractions

Astrocyte-specific mitochondria were isolated from AmtGFP mice using a similar column-based approach. Following brain homogenization and MACS column binding, astrocytic mitochondria were recovered by flushing the column, like the soma fraction and used for downstream application after quantification. Synaptosomal fractions were collected in parallel, pelleted by centrifugation, resuspended in IB, and processed for immunostaining on glass slides to confirm mitochondrial transfer from astrocytes to synaptosomes.

### Mitochondrial bioenergetics measurement:

Following cell type–specific mitochondrial isolation, bioenergetic function was assessed using the Seahorse XFe96 Analyzer (Agilent Technologies, Santa Clara, CA, USA) as previously described [28, 31, 32]. Mitochondrial suspensions were diluted in respiration buffer consisting of 125 mM KCl, 0.1% fatty acid–free BSA, 20 mM HEPES, 2 mM MgCl_2_, and 2.5 mM KH_2_PO_4_, adjusted to pH 7.2 with KOH. Mitochondrial protein was loaded into Seahorse assay plates at 2.5 μg/well for soma fractions and 4 μg/well for total, synaptic, non-neuronal (NN), and astrocytic fractions (3–5 technical replicates per sample). OCR was recorded following sequential injections of substrates, inhibitors, and uncouplers of the mitochondrial electron transport chain (ETC), prepared in respiration buffer lacking BSA. *State III respiration* (ATP-linked respiration): Pyruvate (5 mM) and malate (2.5 mM) were added as Complex Ispecific substrates, followed by ADP (4.3 mM) to stimulate ATP synthase. *State IV respiration* (proton leak respiration): Oligomycin (2.5 μM) was added to inhibit ATP synthase. *State V(CI) respiration* (Complex I–driven uncoupled respiration): FCCP (4 μM), a protonophore uncoupler, was added to collapse the proton gradient and maximize respiration. *State V(CII) respiration* (Complex II–driven uncoupled respiration): Rotenone (0.8 μM) was added to inhibit Complex I, followed by succinate (10 mM) as a Complex II-specific substrate.

### Brain tissue harvesting for single-cell transcriptomics:

At the prescribed interval, mice (n = 2 per group) were anesthetized with 5.0% isoflurane before exsanguination and transcardial perfusion with ice-cold Dulbecco’s phosphate-buffered saline (DPBS; Gibco #14040133). Following perfusion, brain tissues were removed, and the pericontusional cortex or analogous region in sham mice was rapidly dissected.

### Single cell sequencing tissue prep

This dissected tissue from each mouse was immediately transferred into a gentleMACS C-tube (Miltenyi #130-093-237) containing Adult Brain Dissociation Kit (ADBK) enzymatic digest reagents (Miltenyi #130 107 677) prepared according to the manufacturer’s protocol. Tissues were dissociated using the “37C_ABDK” protocol on the gentleMACS Octo Dissociator instrument (Miltenyi #130-095-937) with heaters attached. After tissue digestion, cell suspensions were processed for debris removal following the manufacturer’s suggested ABDK protocol. Following completion of this protocol, cell suspensions were pelleted at 300x*g* for 3 min and gently resuspended in 200 μL of DPBS + 0.4% bovine serum albumin (Invitrogen #AM2616). Cells were sequentially filtered two more times using Flowmi cell strainers (70 μm pore size, Bel-Art #H13680-0040). Cell counts and viability were assessed using AO/PI cell viability dyes (Logos Biosystems #F23001) in tandem with the CellDrop automated cell counter (DeNovix). All samples had > 90% viable cells and were diluted according to 10x Genomics’ suggested concentrations for capturing approximately 20k cells per library (Next GEM 3’ Gene Expression v3).

### Processing of single-cell FASTQ files, dimension reduction, and cell clustering

Pre-processing of scRNAseq data was accomplished using Cell Ranger (v8.0.0, 10x Genomics), with Illumina files aligned via STAR (2.5.1b) to the genomic sequence (introns and exons) using the Mouse (GRCm39) 2024-A annotation. Standard pre-processing protocols were followed in Cell Ranger to identify cells above background and the resulting filtered gene matrices were utilized in Seurat (v5) for single-cell analyses. Genes that were detected in 3 or more cells were used for analyses. Single-cell QC was performed to exclude cells with less than 200 genes or those that exceeded 2500 genes, and greater than 25% mitochondrial genes. Following QC, the two sample objects were merged into a single Seurat object. Data were normalized, scaled, and variable features found using *SCTransform* with default parameters for the merged object. Subsequently, PCA and UMAP were used for two-dimensional visualization of the dimensionally reduced dataset (with the top 30 PCs used, based upon the total variance explained by each). *FindMarkers* function was used to identify the top10 biomarkers for each cell cluster. Cell clusters expressing canonical astrocyte-specific genes (i.e. *Aldoc, Slc2a1, Aqp4)* were identified. These cell clusters were subsequently subsetted and stored as a new object, to which the *SCTransform* and dimension reduction, visualization, and cell identification methods described above were re-applied. After subsetting and data normalization, small groups of distinct clusters with differentially expressed genes (DEGs) corresponding to microglia, ependymal cells, and an unclassifiable group were distinguishable from the main astrocyte groups. Following the removal of these unidentified cells, the remaining astrocyte-selected cells were used for the following downstream analyses, described below.

### Cell type proportion analysis

Cell type proportions were calculated from single-cell metadata by aggregating cell counts by surgical condition (Sham vs CCI) and cluster identity. Pseudocounts of 0.5 were added to avoid zero-inflation issues in compositional data analysis. Bayesian compositional analysis was performed using a Dirichlet regression model implemented in brms v2.22.0 with a logit link function and QR decomposition for numerical stability. MCMC sampling was conducted using 4 chains with 2,000 iterations each. Statistical differences between conditions were assessed using posterior predictions to calculate condition differences (CCI - Sham) for each cell type. Cell types were considered significantly different if the 95% credible interval excluded zero.

### Mitochondrial pathway enrichment analysis

Single-cell gene set enrichment analysis was performed using escape v2.2.3 to evaluate mitochondrial pathway activity across conditions. Mitochondrial gene sets were obtained from MSigDB collections using msigdbr for Mus musculus. Enrichment analysis was conducted using single-sample gene set enrichment analysis (ssGSEA) with normalize = TRUE and a minimum gene set size of 4 genes. Results were visualized using hierarchical clustering of both rows and columns with scaled enrichment scores.

### Differential expression analysis

Differential expression analysis was performed between the CCI and Sham conditions using Seurat’s FindMarkers function with the SCT assay and the default Benjamini-Hochberg multiple testing correction. The Wilcoxon rank-sum test was employed with logfc.threshold = 0 and min.pct = 0 to include all genes for comprehensive analysis. Genes were classified as significantly differentially expressed based on adjusted p-value < 0.001 and absolute log2 fold change > 1. MA plots were generated to visualize the relationship between average expression and log2 fold change.

### Data visualization

Cell type distributions were visualized using stacked bar plots implemented in dittoSeq v1.18.0, displaying proportional representation across experimental conditions and individual samples. Gene expression patterns across clusters were visualized using stacked violin plots implemented in scCustomize v3.1.3.

### Statistics

Statistical analysis was performed using Graph Pad Prism (GraphPad Software, CA, USA). A significant difference among groups was defined as p < 0.05 for all analyses. The Shapiro-Wilk test was completed to ensure normality. As these criteria were met for all experimental data, parametric statistics were employed for all analyses. A two-way ANOVA with Sidak post-hoc multiple comparisons test or one-way ANOVA with Tukey post-hoc multiple comparisons or unpaired t-test used depending upon the groups.

## Results

### Changes in neuronal mitochondrial morphological dynamics impair synaptic mitochondrial function preferentially over soma after TBI:

**Figure 1:**
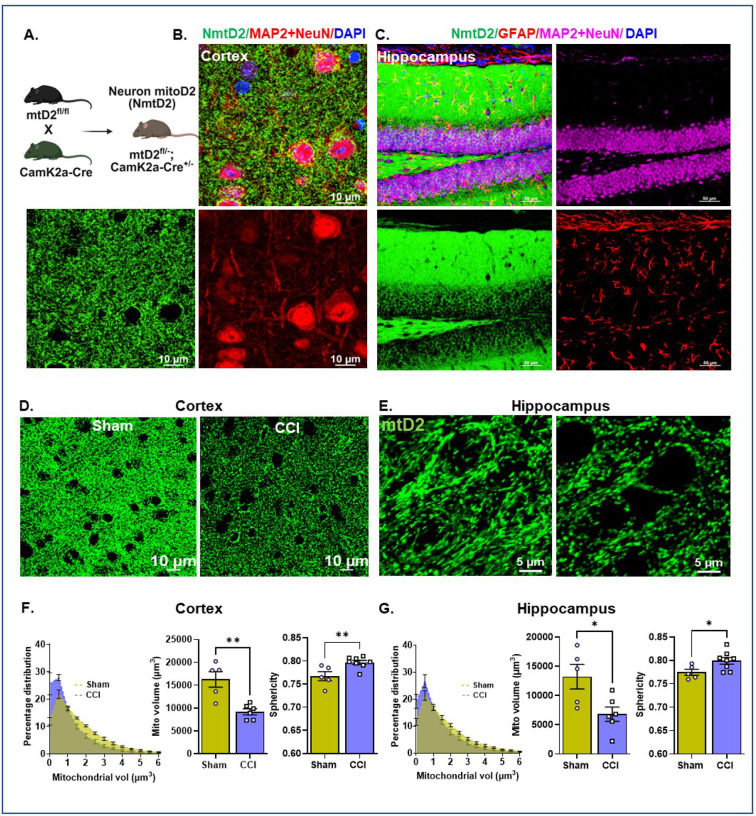
TBI alters neuronal mitochondrial dynamics (A) Schematic illustrating the generation of neuron-specific mitochondrial reporter mice (mtD2^f/f^; CamK2a-Cre^+/−^) expressing the green fluorescent protein Dendra2 (NmtD2) targeted to the inner mitochondrial membrane. (B, C) Representative confocal micrograph of a cortex and hippocampal brain section from NmtD2 mice (mtD2-green), stained for MAP2+NeuN (red/pink), glial fibrillary acidic protein (GFAP-red), and DAPI (blue). (D, E) Representative confocal micrographs of cortex and hippocampus brain sections from NmtD2 mice 24 hrs post-CCI or sham surgery. (F, G) Quantification of mitochondrial morphology using Imaris software from the ipsilateral penumbral cortex and hippocampus regions 24hrs post-injury. The histograms display the percentage distribution of mitochondrial volume, while the bar graphs illustrate the total mitochondrial volume and sphericity. Each circle/square represents one animal. Data represented as Mean ± SEM. P ≤ 0.05 *; P ≤ 0.01** by unpaired t-test.

**Figure 2:**
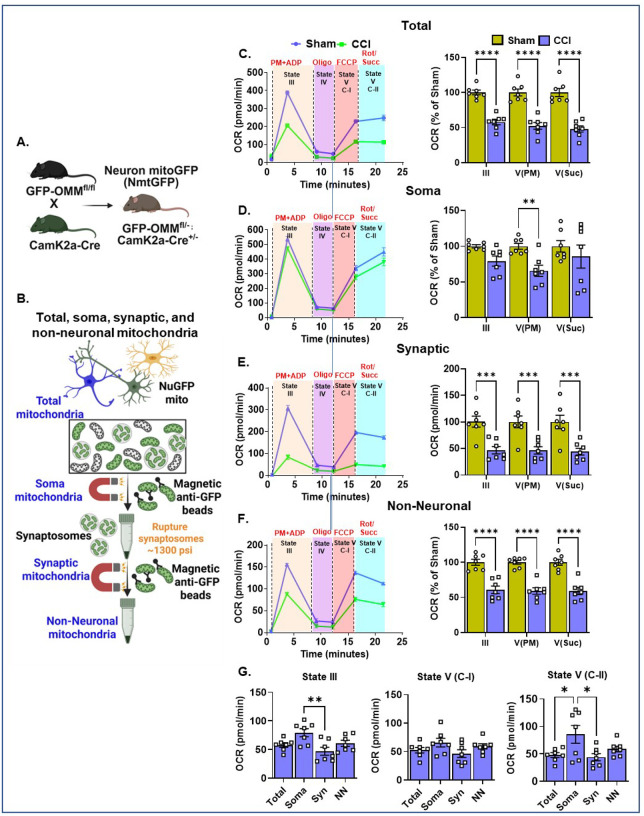
TBI preferentially impairs synaptic mitochondrial function over soma and non-neuronal mitochondrial fraction (A) Schematic illustrating the generation of neuron-specific mitochondrial reporter mice (mtGFP^f/f^; CamK2a-Cre^+/−^) expressing the green fluorescent protein GFP (NmtGFP) targeted to the outer mitochondrial membrane. (B) Flow diagram outlining the isolation of total, neuronal soma, synaptic (Syn), and non-neuronal (NN) mitochondrial fractions from the brain using anti-GFP magnetic beads in NmtGFP mice, 24hrs post-CCI. (C, D, E, F) Representative traces and quantification of OCR (normalized to sham) from the total, neuronal soma, Syn, and NN fraction of mitochondria isolated from sham and CCI (ipsilateral punch). Each circle/square represents one animal (n = 7 mice/group). (G) Bar graphs comparing mitochondrial dysfunction represented as change in OCR (normalized to sham) across four distinct mitochondrial fractions (total, soma, synaptic, and non-neuronal), 24hrs post-CCI; (n=7 mice/group). Data represent mean ± SEM. P ≤ 0.05 *; P ≤ 0.01 **; P ≤ 0.001 ***; P ≤ 0.001 ***; P ≤ 0.0001 **** by two-way ANOVA with Fisher’s LSD comparison test (C, D, E, F); by one-way ANOVA with Tukey post-hoc test (G).

Changes in mitochondrial dynamics and altered expression of fusion and fission proteins are observed in several neurodegenerative diseases [33, 34]. Depending on the context and disease condition, it could be compensatory or detrimental [35–40]. We reported that TBI drastically alters mitochondrial dynamics (size, shape, and count) using mice expressing mtD2 globally [30]. Decades of work from our lab and others have demonstrated that glutamate-mediated excitotoxicity after TBI causes mitochondrial dysfunction through excessive Ca^2+^ influx into neurons [29, 32, 41–44]. However, the specific effects of TBI on neuronal-specific mitochondrial morphology and bioenergetics remain undetermined. To address this, we generated two neuron-specific mitochondrial reporter mouse models: 1. NmtD2, which expresses mtD2 targeted to the inner mitochondrial membrane (Fig. 1A), has better fluorescence, suitable for most of the mitochondrial morphology studies. 2. NmtGFP, which expresses GFP targeted to the outer mitochondrial membrane (Fig. 2A), is suitable for mitochondrial isolation. We validated neuron-specific expression of both reporters by immunostaining brain sections with neuronal markers (NeuN+MAP2) and the astrocytic marker GFAP (Fig. 1B, C). Change in mitochondrial morphology was determined in the penumbral region of injured and sham brain sections, both in cortex and hippocampus (Fig. 1D, E), 24 hrs post-TBI in NmtD2 mice. The percent mitochondrial volume distribution, volume, and sphericity (Fig. 1F, G) in both the cortex and hippocampus demonstrated significantly decreased mitochondrial volume and size, while mitochondrial sphericity increased dramatically after CCI compared to sham. We were curious to see whether the drastic changes in the mitochondrial morphology observed were compensatory or detrimental. To determine this, we isolated neuronal soma, synaptic (Syn), and non-neuronal (NN) fractions of mitochondria using magnetic anti-GFP antibody beads along with total mitochondria from NmtGFP mice (Fig. 2A, B).

The intactness of the isolated mitochondrial membrane potential was tested before running mitochondrial bioenergetics using TMRE and FCCP in mtGFP and WT mitochondria (Fig. S1A). Staining with TMRE and no-TMRE stain in mitochondria with FCCP pretreatment demonstrated the intactness of the anti-GFP-based mitochondrial isolation. Also, no mitochondrial pull-down from WT mice using anti-GFP beads demonstrates the specificity of the GFP beads. The amount of anti-GFP antibody required per gram of tissue, the concentration of necessary mitochondria per well in the Seahorse XF96 plate (Fib. S1B), and the concentration of uncoupler (FCCP) were optimized (Fig. S1C).

Our previous studies demonstrate mitochondrial dysfunction from total mitochondrial fraction after injury in the CCI model[32, 45]. Hence, we used the total mitochondrial fraction as our control to compare the soma, Syn, and NN mitochondrial fractions 24hrs after injury. Results demonstrate that, overall, injury decreased OCR across different states of mitochondrial bioenergetics in all four fractions (Total, soma, Syn, and NN) (Fig. 1C, D, E, and F). There was a significant OCR decrease only at state V(C-I) of the soma fraction (Fig. 1D) compared to all states in the synaptic fraction (Fig. 1E). When comparing only the injured animals with all four different fractions, the synaptic fraction was most affected with the lowest OCR whereas the soma fraction was least affected with the highest OCR (Fig. 1G).

Together, these results demonstrate that changes in neuronal mitochondrial morphology following TBI are detrimental, with decreased mitochondrial bioenergetics observed primarily in the synaptic fraction compared to the soma fractions. Although synaptic mitochondria participate in the synaptic transmission of action potentials, soma mitochondria appear to respire at a higher rate. This likely maintains neuronal homeostasis and makes them less vulnerable to injury compared to synaptic mitochondria.

### TBI increases astrocytic mitochondrial transfer to neuronal soma more than neuronal synaptosomes:

**Figure 3:**
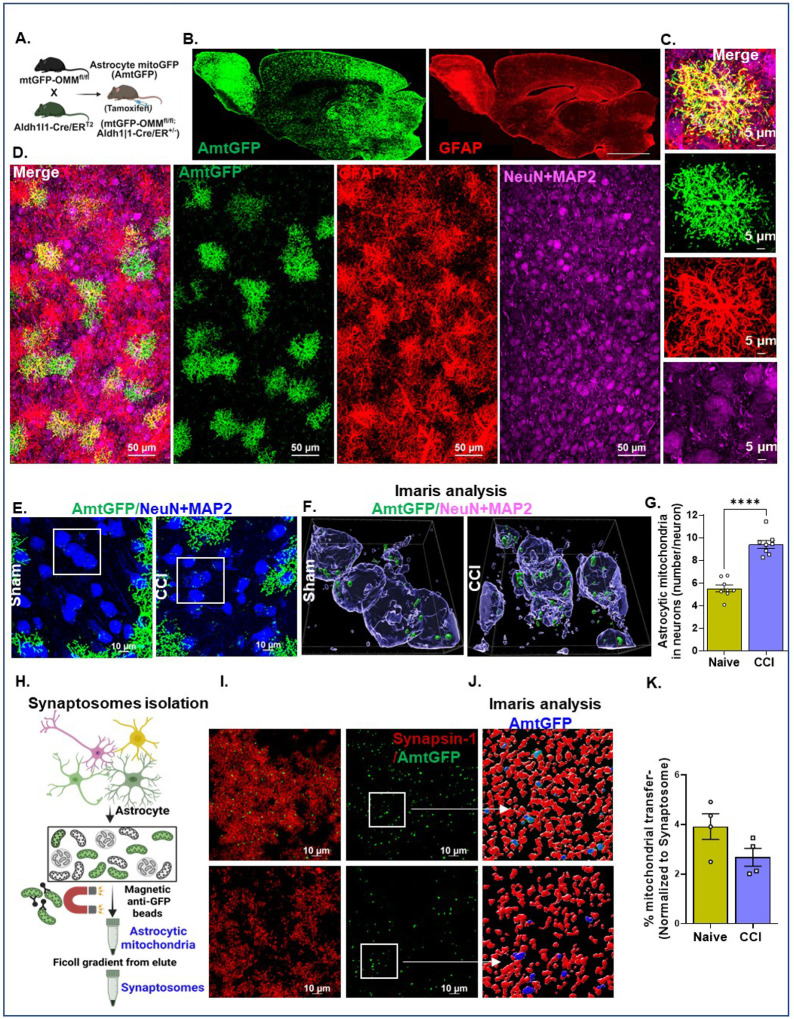
TBI increases mitochondrial transfer from astrocytes to neurons. (A, B) Schematic illustrating the generation of astrocyte-specific mitochondrial reporter mice (AmtD2) and representative micrograph of a brain section from AmtD2 mice (AmtGFP-green; GFAP-red). (C, D) Representative confocal brain sections from AmtD2 mice (AmtGFP-green; GFAP-red; NeuN+MAP2-pink). (E, F) Representative cortical confocal images from naïve and 24 hrs post-CCI AmtGFP mice (E), with 3D Imaris reconstructions (F) illustrating astrocyte-derived mitochondria (green puncta) within neurons (MAP2+NeuN-blue/pink). (G) Quantification of mitochondrial transfer was performed using Z-stack images, which were analyzed in NIS-Elements General Analysis (Nikon) (Fig. S2 for details). Quantification of astrocyte-derived mitochondria per neuron in naïve and 24 hrs post-CCI mice. Data represent eight random, unbiased microscopic fields from 3 naïve mice (139 neurons total) and 4 CCI mice (95 neurons total). (H) Flow diagram outlining the isolation of synaptosomes from AmtGFP mice using Ficoll gradient centrifugation following depletion of astrocyte-specific mitochondria with anti-GFP magnetic beads. (I) Representative confocal micrographs of isolated synaptosomes from naïve and 24 hrs post-CCI AmtGFP mice. (J) Astrocyte-derived mitochondria (green) within synaptosomes (synapsin-1-red) were quantified using confocal Z-stack imaging and 3D-NIS-Elements General Analysis (Nikon). (K) Quantification of normalized percent mitochondrial transfer from astrocytes to synaptosomes, calculated by normalizing astrocyte-derived mitochondria to the total synaptosome number using NIS-Elements analysis; n = 4 mice per group. Data represent mean ± SEM. P ≤ 0.0001 **** by unpaired t-test (H, I).

Astrocytes play a vital role in cerebral metabolism, neurodevelopment, neurotransmission, maintenance of the blood-brain barrier, and blood flow[46]. Under physiological and pathological conditions, excess glutamate released from the presynaptic terminal is cleared by astrocytes, thereby preventing glutamate excitotoxicity and neuronal death [47, 48]. Also, astrocytes support neuronal viability by transferring functional extracellular mitochondria [20, 23, 49, 50]. However, it is not known whether astrocytes transfer mitochondria to rescue or preserve neuronal function after overt pathology, such as TBI. To address this, we used mitochondrial reporter mice generated in our laboratory (AmtD2 and AmtGFP), and we confirmed astrocyte-specific mitochondrial reporter gene expression in brain sections (Fig. 3A, B, C, D, and Fig. S2A). Naïve mouse brain sections from AmtD2, stained with neuronal markers (NeuN+MAP2) and astrocyte marker (GFAP), were used to assess mitochondrial transfer from astrocytes to neurons under physiological conditions. 3D reconstruction from confocal z-stack images in Imaris software enabled the visualization of astrocytic mitochondria in neurons (Fig. 2B; Video1). Importantly, we detected astrocytic mitochondria in proximity to neuronal nuclei using 3D Imaris (Fig. S2B). This observation eliminates ambiguity about whether they are just juxtaposed on top of the neurons, not getting inside.

Next, we examined whether TBI affects astrocyte-to-neuron mitochondrial transfer using AmtGFP mice. We selected this reporter line because it provides superior separation of astrocytic and neuronal mtGFP signals compared to AmtD2 mice, enabling unambiguous and unbiased quantification of transferred mitochondria. Brain sections from the penumbral cortex of injured and naïve mice were stained with neuronal markers (NeuN and MAP2) (Fig. 3E). Using 3D-rendered Imaris reconstructions, we visually detected astrocyte-derived mitochondria within neuronal somata after TBI (Fig. 3F). Defining neurons as the region of interest (ROI), we quantified astrocytic mitochondrial transfer using the unbiased pipeline described in Fig. S3 A, B. TBI significantly increased astrocyte-derived mitochondrial transfer to neurons compared to naïve controls (Fig. 3G). To further characterize astrocyte-derived mitochondria at synaptic compartments, we isolated synaptosomes from the cortex of AmtGFP mice (Fig. 3H) and stained them with the synaptic marker synapsin-1 (Fig. 3I). Overall, synaptosome abundance was reduced after TBI relative to naïve animals. However, when normalized to total synaptosome number using 3D-NIS elements general analysis (Fig. 3J), the proportion of synaptosomes containing astrocyte-derived mitochondria did not differ significantly between TBI and naïve groups (Fig. 3K).

Interestingly, this pattern suggests a correlation between astrocytic mitochondrial transfer and neuronal mitochondrial function. When astrocyte-derived mitochondria did not increase at synapses after TBI, synaptic mitochondrial dysfunction was pronounced. Conversely, in neuronal soma, where mitochondrial transfer was elevated, mitochondrial dysfunction appeared less severe. Together, these findings support the possibility that astrocytic mitochondrial donation acts as a compensatory mechanism to preserve neuronal function following TBI.

### TBI does alter mitochondrial morphology but not astrocytic mitochondrial bioenergetics:

**Figure 4:**
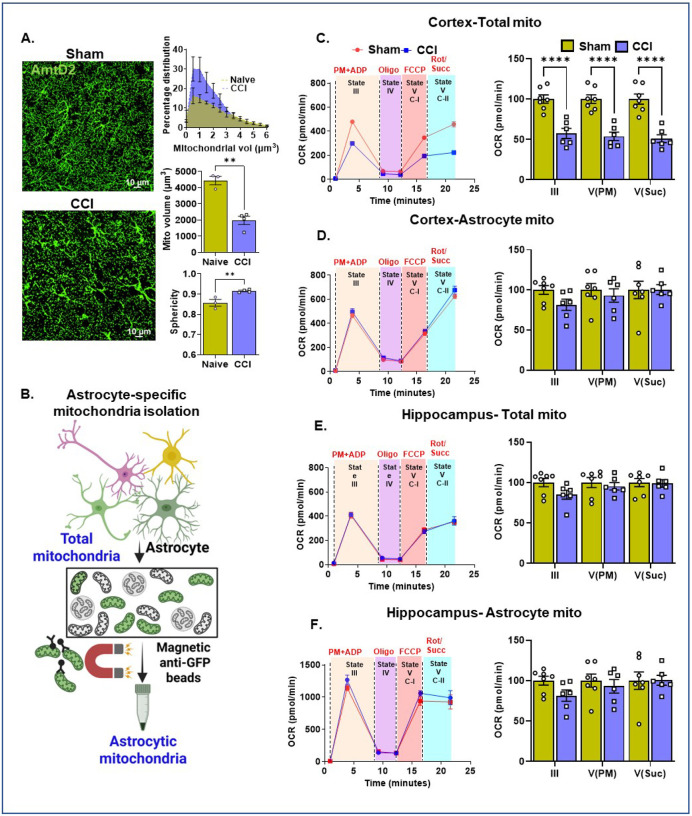
TBI does not affect astrocytic mitochondrial bioenergetics but alters dynamics. (A) Representative confocal micrographs of cortical sections from sham and 24hrs post-CCI AmtD2 mice. Quantification of cortical astrocytic mitochondrial percentage distribution, volume, and sphericity (D), measured from confocal Z-stack images using Imaris. Each circle or square represents one animal (n = 3–4 mice/group). (B) Schematic of the workflow of isolating astrocyte-specific mitochondria using anti-GFP magnetic beads from AmtGFP mice. (C, D) Representative OCR traces and quantification of isolated total (C) and astrocytic (D) mitochondrial fractions from the cortex of sham and CCI mice (ipsilateral punches). Each circle or square represents one animal (n = 6–7 mice/group). (E, F) Representative OCR traces and quantification of isolated total (E) and astrocytic (F) mitochondrial fractions from the hippocampus of sham and CCI mice (ipsilateral punches). Each circle or square represents one animal (n = 6–7 mice/group). Data represent mean ± SME. P > 0.05, non-significant (ns); P ≤ 0.01 ** by unpaired t-test (A); by two-way ANOVA with Fisher’s LSD post hoc comparison (C,

Given that changes in mitochondrial morphology after TBI were associated with reduced neuronal mitochondrial function and that we concurrently observed increased astrocyte-derived mitochondrial transfer to neurons, we next asked whether astrocyte mitochondrial morphology changes after injury and whether astrocytes transfer respiratory-competent mitochondria following injury. To test this, astrocytic mitochondrial morphology was first assessed in the penumbral regions of the cortex and hippocampus of AmtD2 mice 24hrs after injury. In the cortex, a significant decrease in the percentage distribution of mitochondrial volume and total volume, and an increase in sphericity, indicate a fission-like phenotype, reflecting mitochondrial fragmentation. In contrast, mitochondrial morphology in the hippocampus did not show similar alterations after injury (Fig. 4A).

Next, we isolated astrocyte-specific mitochondrial fractions from the cortex and hippocampus of AmtGFP mice (Fig. 4B) and assessed their bioenergetics. Control experiments with the total mitochondrial fraction demonstrated mitochondrial dysfunction in the cortex (Fig. 4C) and not in the hippocampus (Fig. 4E), as expected. Surprisingly, astrocytic mitochondria from both the cortex and hippocampus maintained normal mitochondrial function 24hrs after TBI, with no reductions in respiratory capacity compared to naïve controls (Fig. 4D, F). These results suggest that although both neurons and astrocytes respond to TBI by altering mitochondrial dynamics, only neuronal mitochondria become dysfunctional at this early time point, whereas astrocytic mitochondria remain intact. This distinction supports a model in which excitotoxic activation of NMDA and glutamatergic signaling leads to Ca^2+^ overloading and mitochondrial injury in neurons, while simultaneously, astrocytes may transfer healthy, respiratory-competent mitochondria to neurons as a compensatory rescue mechanism.

### TBI alters gene expressions associated with intercellular communication and mitochondrial translation machinery in astrocytes after CCI:

Astrocytes became activated within 24hrs following CCI model of TBI, as demonstrated by immunofluorescence analysis of glial fibrillar acidic protein (GFAP) expression (Fig. 4A). GFAP staining revealed a significant increase in both expression levels and coverage area in the injured brain compared to sham controls (Fig. 4B). Reactive astrocytes are known to play a dual role in the pathophysiology of TBI. On one hand, depletion of reactive astrocytes can exacerbate inflammation and promote neuronal death [51]. On the other hand, in transgenic models lacking reactive astrocyte markers such as vimentin and GFAP, neuronal repair mechanisms are enhanced[52, 53]. In our experimental conditions, TBI increased astrocyte-to-neuron mitochondrial transfer without altering the functional integrity of the transferred mitochondria. To investigate the mechanisms underlying astrocyte-mediated mitochondrial transfer after TBI, we performed single-cell RNA sequencing (scRNA-seq) of astrocytes isolated 24hrs post-injury. TBI induced a profound remodeling of the astrocyte landscape, shifting the population from predominantly homeostatic astrocytes (As-1 and As-2) toward injury-associated subpopulations (As-4 to As-8) (Fig. 4C,D). Violin plot analysis revealed increased expression of reactive astrocyte markers (Lcn2, Gfap, Serpina3n, and Vim) within injury-associated clusters, while homeostatic markers (Aldoc, Slc1a3, and Gja1) remained enriched in As-1 and As-2 (Fig. 4E). Pathway enrichment analysis demonstrated that homeostatic astrocytes were enriched for mitochondrial biogenesis, oxidative phosphorylation, TCA cycle activity, and mitochondrial fusion, whereas injury-induced clusters, particularly As-7 and As-8, were enriched for extracellular vesicle (EV) biogenesis, mitophagy, and PGC1α signaling pathways (Fig. 4F). Consistent with these findings, MA plot identified significant upregulation of genes involved in EV biogenesis and cargo loading (Sdc4, Cd9, Cd63), EV formation and secretion (Sdcbp), TNT formation and cytoskeletal remodeling (Tuba1a, Flna, Fscn1, Actr3), and mitochondrial translation (Mrps6, Mrpl33) (Fig. 4G). Collectively, these data suggest that neuronal mitochondrial dysfunction following TBI drives astrocytes toward a reactive, mitochondria-supportive phenotype characterized by enhanced mitochondrial quality control and intercellular trafficking pathways, thereby promoting the transfer of respiratory-competent mitochondria to injured neurons as an endogenous neuroprotective response.

### Exogenous Ast-EV-mito treatment reduces excitotoxicity-induced mitochondrial dysfunction in primary neurons:

Currently, mitochondrial transfer between cells is considered a protective or rescue mechanism [22, 27, 54–60]. This phenomenon is being explored as a treatment option through exogenous mitochondrial transplantation for several diseases, notably neurodegenerative diseases [25]. However, several open questions remain in the field of mitochondrial transplantation. What is the fate of transplanted mitochondria? Are they getting integrated along with the host mitochondria? Do they maintain or improve the mitochondrial function of the host cell? Previous studies, including our own, have demonstrated the feasibility of exogenous mitochondrial transplantation [20, 22, 61]. However, there is no direct evidence to suggest that excitotoxicity-mediated mitochondrial dysfunction can be rescued by mitochondrial transplantation.

**Figure 6:**
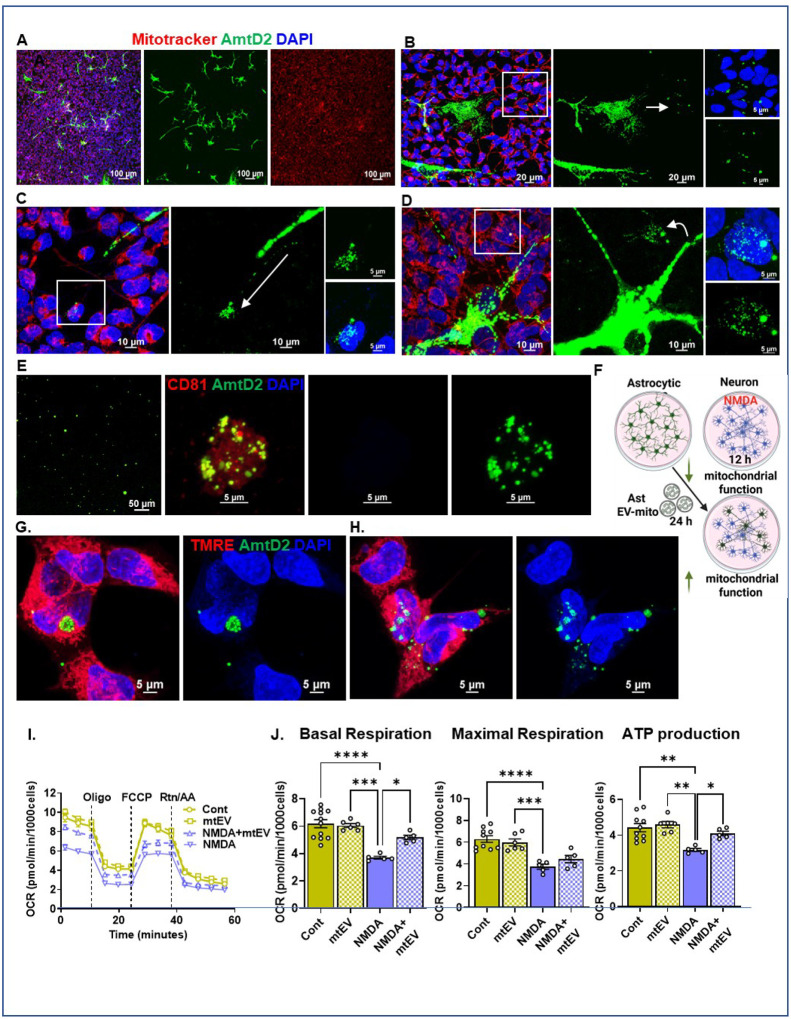
Astrocytic EV-mito improves neuronal mitochondrial function following NMDA-mediated excitotoxicity via mitochondrial transfer (A, B, C, D) Representative confocal micrograph of neuron (SH-SY5Y) - astrocyte (AmtGFP) cell co-culture showing mitochondrial transfer (White arrows) from astrocytes to neurons by TNT (C) and EV-mito (B, D). AmtGFP astrocyte images were over-saturated to see neuronal mitochondrial transfer due to the lower fluorescence intensity of a few transferred mitochondria. Mito-transferred cells zoomed in subsets (white square). AmtGFP-green; Mitotracker-red; DAPI-blue. (E) Representative micrographs of isolated astrocyte-derived EV-mito from AmtD2 primary astrocyte conditioned media stained with CD81 (EV marker). DAPI-blue; CD81-red; AmtGFP-green. (F) Schematic outlining of the experimental paradigm for astrocytic EV-mito transplantation after NMDA treatment in primary cortical neurons. (G, H) Representative confocal micrograph of neurons (SH-SY5Y) after treatment with astrocytic EV-mito, show internalized astrocytic EV-mito visible within the neuron (G) and distributed within the cells (H). Endogenous mitochondria were trained with Mitotracker-red. DAPI-stained nuclei (blue). (I) Representative oxygen consumption rate (OCR) traces from mitochondrial stress tests (MST) in neurons treated with Control, EV-mito, NMDA+EV-mito, or NMDA alone. Sequential injections of oligomycin, FCCP, and rotenone/antimycin A are indicated. (J) Quantification of basal respiration, maximal respiration, and ATP production derived from Seahorse XFe96 analysis. Data represent n = 5–12 technical replicates per group, repeated in two independent experiments with similar results. Data represent mean ± SEM. P ≤ 0.05 *; P ≤ 0.01**; P ≤ 0.001***; P ≤ 0.0001**** by unpaired t-test (B); by one-way ANOVA with Tukey’s multiple comparisons test (I).

To visualize mitochondrial transfer, we co-cultured SH-SY5Y cells with AmtD2 primary cells (Fig. 6A) or SH-SY5Y cells treated with EV-mito isolated from AmtD2 conditioned media (Fig. 6G). Co-culture experiments demonstrate that mitochondrial transfer from the astrocytes to neurons through EV-mito transmission (Fig. 6B, D) or TNTs (Fig. 6C). We tested for the presence of mtD2 fluorescence in astrocyte derived EV-mito using EV marker (CD81) (Fig. 6E). We were able to visualize transplanted astrocyte-derived EV-mito inside the neuronal cell (Fig. 6G), released inside the cells co-exist with endogenous neuronal mitochondria (stained with Mito Tracker-Red) (Fig. 6H). To test the rescue effect of astrocyte derived EV-mito, NMDA pretreated cortical primary neurons were transplanted with E-mito (Fig. 6F). The MST revealed that EV-mito transplantation significantly increased basal respiration and ATP production in NMDA-treated primary neurons, but not in the untreated group (Fig. 6I and J). Collectively, these data indicate that healthy neurons can take up exogenous EV-mito and may not necessarily utilize them for respiration. Alternatively, dysfunctional neurons may use the transplanted exogenous mitochondria for their aerobic respiration and survival, consistent with previous studies in macrophages [61].

## Discussion

Our study identifies astrocytic mitochondrial transfer as a fundamental mechanism of neuronal protection after TBI, with implications that extend beyond injury to broader principles of glial-neuronal metabolic coupling. Although TBI profoundly alters mitochondrial dynamics in both neurons and astrocytes, the most profound functional deficits occur in neuronal mitochondria, particularly within synaptic compartments that are highly energy-dependent. Astrocytes, in contrast, preserve mitochondrial respiratory capacity and activate transcriptional programs related to organelle trafficking, extracellular vesicle biogenesis, and mitochondrial translation, thereby positioning themselves as effective donors of mitochondria. Consistent with these transcriptional changes, we observed enhanced astrocytic mitochondrial transfer to neurons *in vivo* following TBI. Mechanistically, we further demonstrate that astrocyte derived EV-mito were sufficient to restore mitochondrial function in primary cortical neurons exposed to NMDA, directly linking mitochondrial transfer to neuronal resilience. Together, these findings highlight a previously underappreciated role of astrocytes in dynamically supporting neuronal survival through organelle donation, suggesting that harnessing mitochondrial transfer may represent a therapeutic strategy for neurodegenerative and neurovascular disorders characterized by mitochondrial dysfunction.

### Contrasting neuron and astrocytic mitochondrial bioenergetics following TBI

Currently, brain mitochondrial isolation from our lab and others follows either ficoll/Percoll-based isolation [9, 62, 63] or TOM22 antibody-based Fractionated mitochondrial magnetic separation (FMMS) [31]. Both of these methods give rise to two distinct populations of mitochondria, namely, synaptic and non-synaptic. The non-synaptic fraction of mitochondria contains neuronal soma and mitochondria from all other brain cell types, while synaptic mitochondria are isolated from neuronal synaptosomes using high-pressure (~ 1200 psi) rupture, followed by ficoll/percoll purification. It was impossible to isolate cell-type-specific mitochondria until the Misgeld laboratory generated Mito Tag mice, which express a Cre recombinase-dependent GFP protein targeted to the outer mitochondrial membrane (OMM)[64]. Using a GFP antibody, they isolated cell-specific mitochondria from cerebellar Purkinje cells, granule cells, and astrocytes, and determined the mitochondrial proteomic diversity. However, to date, no one has studied cell-type-specific mitochondrial bioenergetics in the brain. In this study, we developed four different mouse models to investigate mitochondrial bioenergetics and mitochondrial morphology in neurons and astrocytes following brain injury.

Mitochondrial dynamics are strongly associated with mitochondrial bioenergetics [34, 65–68]. Increased mitochondrial fission is associated with extended physiological demand [69]. However, chronic mitochondrial fission drives pathology and mitochondrial dysfunction [33, 34, 70, 71]. TBI is associated with acute mitochondrial fission [30]. However, cell specificity and associated bioenergetics are not well understood. In our study using NmtD2 and AmtD2 mouse models (expressing Dendra2 from the inner mitochondrial membrane), we confirmed that both neurons and astrocytes respond to injury, exhibiting an increased mitochondrial fission-like phenotype associated with a change in morphology towards a spherical rather than slender shape. To study cell-specific mitochondrial bioenergetics, we used NmtGFP and AmtGFP mouse models (which express GFP from the outer mitochondrial membrane).

From NmtGFP, we isolated four different fractions of mitochondria, namely total, soma, synaptic, and NN, using an anti-GFP antibody. Bioenergetics measurements reveal that out of these four fractions of mitochondria, only synaptic mitochondria were most vulnerable to TBI. This result is consistent with previous studies that used the Ficoll/Percoll method to isolate the synaptic fraction of mitochondria[7, 63, 72, 73]. In our method, we further separated neuronal soma and the NN mitochondrial fraction with one more round of anti-GFP antibody. Interestingly, we found the least mitochondrial dysfunction from the soma mitochondrial fraction after injury compared to other fractions. Still, the NN fraction (glial-enriched) cells showed significant mitochondrial dysfunction. To study further, we used AmtGFP mice to isolate only astrocyte-specific mitochondria from the glial cell population. Bioenergetics data indicated that astrocytic mitochondria had no injury effect in either the cortex or hippocampus. In contrast, the same samples exhibited significant mitochondrial dysfunction from the total mitochondrial fraction.

Taken together, these findings demonstrate that although mitochondrial morphology and dynamics are altered in both neurons and astrocytes following TBI, these changes do not directly correlate with bioenergetic dysfunction at 24hrs post-injury. While neuronal mitochondrial fractions exhibited significant respiratory deficits, astrocytic mitochondrial bioenergetics remained largely preserved despite injury-induced morphological remodeling. This preservation of mitochondrial function suggests that astrocytes may serve as a source of respiratory-competent mitochondria that can be transferred to neurons to support metabolic recovery following TBI. Future studies utilizing newly generated microglia- and oligodendrocyte-specific mitoGFP reporter mice will further elucidate how TBI affects mitochondrial function and dynamics across distinct non-neuronal cell populations and determine their potential contributions to intercellular mitochondrial transfer within the injured CNS.

### Mitochondrial transfer from astrocytes to neurons, both in vivo and in vitro

There is limited *in vivo* evidence supporting the transfer of mitochondria from astrocytes to neurons, with most studies conducted *in vitro*. Mitochondrial transfer between astrocytes and neurons has been shown to occur via CD38/cyclic ADP-ribose (cADPR) signaling and is regulated by mitochondrial Rho GTPases (MIRO1 and MIRO2). This process is disrupted by mutations in GFAP, as seen in Alexander disease, which impairs astrocyte-to-neuron mitochondrial transfer [74]. Activation of astrocytes with cADPR increases the release of extracellular mitochondrial particles into the media, and the addition of astrocyte-conditioned media to neurons subjected to oxygen-glucose deprivation (OGD) improves ATP levels and neuronal viability, suggesting a neuroprotective role for astrocyte-derived mitochondria [20]. We were the first to demonstrate intercellular mitochondrial transfer from astrocytes to brain capillaries under physiological conditions using a transgenic mouse model expressing a mitochondria-targeted fluorescent reporter. This transfer was observed in naïve mice that was further enhanced with aging[22].

In a related study, a similar approach was employed using an adeno-associated virus (AAV) system to target astrocytes, showing that low-density lipoprotein receptor-related protein 1 (LRP1) facilitates astrocyte-to-neuron mitochondrial transfer. This occurs by downregulating glucose uptake, glycolysis, and lactate production, thereby reducing ADP-ribosylation factor 1 (ARF1) lactylation and promoting mitochondrial transfer [24]. Collectively, these findings support the existence of astrocyte-to-neuron mitochondrial transfer as a physiologically relevant and regulated process. To date, no studies have directly examined how astrocyte-to-neuron mitochondrial transfer contributes to recovery in overt injury models such as TBI. Consistent with previous findings, in this study, we observed that mitochondrial transfer from astrocytes to neurons occurs under physiological conditions, without external stimulation, both *in vivo* and *in vitro*. Notably, this transfer was significantly enhanced following TBI, coinciding with astrocyte activation. Interestingly, our results contrast with a previous study in a mouse stroke model, where inhibition of astrocyte activation using Ginsenoside Rb1 enhanced mitochondrial transfer *in vitro*. This effect was attributed to reduced mitochondrial complex I activity in astrocytes under conditions of the oxygen–glucose deprivation and reperfusion (OGD/R) model *in vitro*[75]. The discrepancy between studies may stem from differences in experimental design. Specifically, the prior study quantified mitochondrial content indirectly by measuring ATP levels in astrocyte-conditioned media, rather than assessing direct intercellular transfer using co-culture or *in vivo* models. Taken together, this study indicates that brain injury enhances astrocyte-to-neuron mitochondrial transfer, likely mediated by glutamate-induced calcium signaling in astrocytes.

### Activated astrocytes are reprogrammed to generate more TNTs, EVs, and mitochondrial biogenesis.

Under physiological conditions, white matter astrocytes and astrocyte end feet surrounding large penetrating vessels exhibit prominent GFAP expression[76]. The functional significance of GFAP is further supported by evidence showing that GFAP deletion impairs reactive astrogliosis[21] increases mortality following TBI [77], and mutations in GFAP associated with Alexander disease impair astrocyte-to-neuron mitochondrial transfer [74]. Both GFAP and vimentin are essential to initiate reactive astrogliosis after TBI to clear debris. However, the precise contribution of GFAP upregulation in activated astrocytes during TBI remains poorly understood. Our results demonstrate that activated astrocytes undergo reprogramming, characterized by altered gene expression associated with mitochondrial biogenesis, mitophagy, TNT formation, EV biogenesis, cargo loading, and secretion. Astrocytes are known to transfer cellular contents to neurons *in vivo* through TNTs or EVs[78–80]. In humans, astrocyte-derived EVs (ADEVs) are increased in plasma one month after stroke [81], and ADEVs isolated from human activated astrocytes have been shown to enhance neuronal uptake, modulate differentiation, and influence neuronal firing[80]. Collectively, these findings suggest that during the acute phase of TBI, astrocytes adopt a specialized neuroprotective phenotype characterized by enhanced mitochondrial quality control and intercellular communication pathways. This adaptive response likely enables astrocytes to generate, traffic, and recycle mitochondria through coordinated mitochondrial transfer and mitophagy mechanisms, thereby supporting neuronal recovery in the injured brain.

### Therapeutic feasibility of Ast-EV-mito transfer/transplantation for excitotoxicity:

Recently, EMT has emerged as a promising therapeutic strategy for diseases characterized by mitochondrial dysfunction, including cardiovascular, respiratory, and neurodegenerative disorders[82–85]. The transfer of healthy mitochondria appears to be an effective rejuvenation process in damaged cells [85, 86]. Previous studies have shown that EMT using EV-mito improves neuronal ATP levels and viability *in vitro* [20]. However, it remains unknown whether EV-mito can mitigate excitotoxicity-induced neuronal dysfunction, a common pathological mechanism underlying several neurodegenerative diseases, including TBI.

In this study, we established that NMDA-mediated excitotoxicity significantly impairs mitochondrial function and alters mitochondrial dynamics in neurons (Fig. 6). Remarkably, we were able to rescue this mitochondrial dysfunction by transplanting healthy mitochondria isolated from astrocyte-conditioned media. These mitochondria, Ast-EV-mito, were endogenously labeled with a mitochondrial-targeted fluorescent reporter (mtD2), which eliminates concerns associated with nonspecific signals due to dye leakage, a common limitation in mitochondrial transplantation studies using exogenous dyes, such as MitoTracker. Furthermore, using high-resolution confocal microscopy coupled with Imaris 3D reconstruction, we demonstrated that transplanted mitochondria not only internalize into recipient neurons but also colocalize with the endogenous mitochondrial marker TOM20, confirming successful integration rather than surface adherence. To directly assess the functional impact of mitochondrial transplantation, we measured neuronal bioenergetics using the Seahorse XF flux analyzer, thereby avoiding the limitations of indirect colorimetric ATP and cell viability assays. Taken together, this study is the first to demonstrate the therapeutic potential of Ast-EV-mito in restoring neuronal mitochondrial function under excitotoxic conditions, a pathophysiological mechanism common to neurodegenerative diseases, including TBI.

### Limitations of the study:

While this study provides novel insights into astrocyte-mediated mitochondrial transfer and its potential therapeutic implications following TBI, several limitations should be acknowledged. First, the analysis was conducted at a single post-injury time point, which may not capture the full temporal dynamics of mitochondrial transfer and recovery processes. Second, although the study focused on astrocyte-derived mitochondria, contributions from other glial or neuronal sources cannot be completely excluded. Similarly, the mitochondrial transfer to cell types other than neurons. Third, while *in vitro* and *in vivo* data support the functional integration of transferred mitochondria, additional studies using high-resolution live imaging and genetic labeling are needed to confirm long-term mitochondrial incorporation and bioenergetic impact in recipient neurons. Furthermore, as with any experimental models, they may not fully replicate the complexity of human TBI pathology, and extrapolation to clinical settings should be approached with caution. Finally, while astrocyte-derived EV-mito shows therapeutic promise, the precise mechanisms regulating mitochondrial uptake, trafficking, and sustained function in injured neurons remain to be fully elucidated.

## Conclusion

Our findings demonstrate that TBI induces a shift in astrocyte phenotype that promotes mitochondrial packaging and transfer to other cells. Astrocytic mitochondrial transfer increased mitochondrial transfer to the neuronal soma but not to synaptic compartments after TBI. This compartment-specific pattern of transfer aligns with our functional data: neuronal soma receiving astrocytic mitochondria maintained mitochondrial bioenergetics and preserved respiratory complex protein levels, whereas synapses, where no increase in astrocytic mitochondrial delivery was detected, exhibited pronounced mitochondrial dysfunction after TBI. These results suggest that insufficient mitochondrial support at synapses contributes to excitotoxic injury and impaired synaptic metabolism. Whether intrinsic proteomic changes within synaptic or somatic mitochondrial populations underlie this differential vulnerability remains an important question for future investigation. Importantly, we observed that astrocytes can release EV-mito, and our proof-of-concept *in vitro* experiments demonstrate that EV-mito supplementation enhances mitochondrial transfer and improves neuronal bioenergetics in an NMDA-induced excitotoxicity model. Together, these findings identify EV-mito as a promising therapeutic strategy to specifically target synaptic mitochondrial deficits that arise following TBI and excitotoxic signaling.

## Supplementary Material

Supplementary Files

This is a list of supplementary files associated with this preprint. Click to download.

- Supplementaryfigures.docx

## Figures and Tables

**Figure 5:**
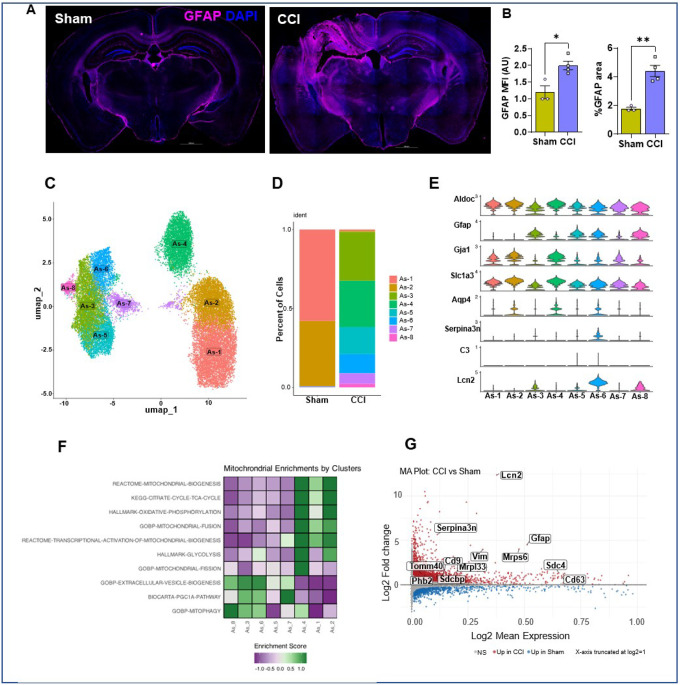
TBI alters gene expressions associated with EV biogenesis and mitochondrial translation machinery in astrocytes after CCI (A) Representative micrographs of whole-brain sections from sham and CCI mice stained with GFAP (astrocytes-pink) and DAPI (nuclei-blue). (B) Quantification of astrocytic mean fluorescence intensity (MFI) and percentage GFAP+ area from brain sections using NIS-Elements General Analysis. Each circle or square represents one animal (n = 3–4 mice/group). (C) UMAP visualization of single-cell transcriptomic profiles from sham and CCI mice (n = 2/group), identifying eight major cell clusters based on canonical marker expression. (D) Proportion of each identified cell cluster represented as a percentage of total cell count in stacked bar plots. (E) Expression patterns of genes associated with reactive astrocytes shown as stacked violin plots across the identified clusters. (F) Astrocyte mitochondrial pathway enrichment analysis displayed as a hierarchically clustered heatmap with scaled enrichment scores. (H) MA plot showing significantly differentially expressed genes, illustrating the relationship between mean gene expression and log2 fold change. Key genes associated with reactive astrocytes, EV biogenesis and cargo loading, tunneling nanotube (TNT) formation and cytoskeletal remodeling, mitochondrial ribosomal proteins, and mitophagy are highlighted.

**Table 1:** 

S.No	Primary antibody	Vendor	Catalogue number	Dilution
1	TOM-20	Cell Signaling	42406S	1:250
2	GFAP	Cell Signaling	80788	1:250
3	RBFOX3/NeuN	Novus biologicals	NBP1-92693	1:250
4	MAP-2	Thermofisher	MA1-25044	1:250
5	β3-Tubulin	Sigma	T2200	1:250
6	NeuN	Cell Signaling	D4G4O	1:250
	**Secondary antibody**			
1	Alexa Flour 488 goat anti-mouse	Thermofisher	A11001	1:500
2	Alexa flour 594 donkey anti-mouse	Thermofisher	A-21203	1:500
3	Alexa flour 594 donkey anti-rabbit	Thermofisher	A21207	1:500
4	Alexa flour 488 donkey anti-rabbit	Thermofisher	A-21206	1:500

## Data Availability

All data supporting the findings of this study are available within the main article and its Supplementary Information files. The single-cell RNA sequencing datasets generated and analyzed during the current study are available from the corresponding author upon reasonable request. Additional source data supporting the figures and conclusions of this study are available from the corresponding author upon request.
